# Exploring the Challenges and Solutions in Conducting Clinical Trials in Saudi Arabia: A Qualitative Study Perspective

**DOI:** 10.3390/healthcare12212182

**Published:** 2024-11-01

**Authors:** Basim Alsaywid, Dana Khafagi, Alaa Bashaikh, Abdulaziz Alsada, Reema Bawazir, Rasal Alotaibi, Lina Alharbi, Rama Alqarni, Rana Alhuthayfi, Hana Alhumud, Shadell AlGhamdi, Mohamed Anwar Khan, Wadha Alshali, Eman Al Mutairi, Miltiadis D. Lytras

**Affiliations:** 1 Education and Research Skills Directory, Saudi National Institute of Health, Riyadh 12382, Saudi Arabia; halhumud@snih.gov.sa (H.A.); walsahli@snih.gov.sa (W.A.); ealmutairi@snih.gov.sa (E.A.M.); 2 Pediatric Urology Section, Department of Surgery, College of Medicine, King Saud University Medical City, King Saud University, Riyadh 11421, Saudi Arabia; 3 Urology Department, Dr. Soliman Fakeeh Hospital, Riyadh 11421, Saudi Arabia; 4 Radiology Department, College of Applied Medical Sciences, University of Jeddah, Jeddah 21959, Saudi Arabia; danakhafaji96@gmail.com; 5 CTI Clinical Trial and Consulting Services, Riyadh 21959, Saudi Arabia; alaa.bashaikh@gmail.com; 6 College of Pharmacy, King Saud University, Riyadh 11451, Saudi Arabiabawazeer.reema@gmail.com (R.B.); 7 College of Nursing, Jazan University, Jazan 45142, Saudi Arabia; rasalalotaibi77@gmail.com; 8 College of Medicine, University of Jeddah, Jeddah 21959, Saudi Arabia; lina_al-harbi@outlook.com; 9 College of Nursing, King Saud Bin Abdulaziz University for Health Sciences, Ministry of National Guard, Health Affairs, Jeddah 11426, Saudi Arabia; ramaalqarni04@gmail.com; 10 Faculty of Medicine, King Abdulaziz University, Jeddah 21551, Saudi Arabia; ralhuthayfi@gmail.com; 11 College of Medicine, King Saud Bin Abdulaziz University for Health Sciences, Ministry of National Guard, Health Affairs, Jeddah 11426, Saudi Arabiakhana@ksau-hs.edu.sa (M.A.K.); 12 King Abdullah International Medical Research Center (KAIMRC), Jeddah 11426, Saudi Arabia; 13 Effat College of Engineering, Effat University, Jeddah 21478, Saudi Arabia

**Keywords:** clinical trials, Saudi Arabia, challenges, solutions, national strategies, initiatives, research education, competency, skillsets, SNIH, qualitative study, thematic analysis, capacity building

## Abstract

Introduction: Clinical trials are crucial for advancing medical knowledge and improving healthcare outcomes. Despite an increase in research publications in Saudi Arabia, clinical trial productivity has lagged behind. Understanding the barriers to conducting clinical trials in Saudi Arabia is essential. This study aims to explore the challenges and propose solutions for improving clinical trial capacity in the Kingdom. Specifically, we aim to develop national strategies to enhance clinical trial infrastructure, identify educational needs, and suggest ways to strengthen research education and training in Saudi Arabia. Methods: The study utilized a qualitative research design with a grounded theory approach. Participants were recruited through purposive sampling, including clinical trial professionals from Riyadh, Jeddah, and Al Khobar, who participated in three half-day workshops. Discussions focused on required skillsets, barriers to conducting trials, improvement recommendations, gaps in research education, and strategies for the Saudi National Institute of Health (SNIH) to support clinical trials. Data were collected through pre-workshop surveys and focus group discussions, and thematic analysis was employed to identify common themes. Results: A total of 35 participants, mainly from the government sector (67%), attended the workshops. Physicians represented the largest professional group (31%), with the majority being Saudi nationals (83%) and engaged in clinical practice (65%). The study identified key challenges, including limited resources (82%), inadequate infrastructure (82%), time constraints (80%), and insufficient funding (80%). While satisfaction with educational programs was mixed, a need for improved infrastructure and support for clinical trials in Saudi Arabia was evident. The focus group discussions further highlighted the importance of research competency, identifying 11 essential domains such as research methodology, data management, and project leadership. Nine major challenges were noted, including funding, infrastructure, and collaboration deficits. A total of 38 recommendations were proposed to address these challenges and enhance the national clinical trial ecosystem. Conclusions: This study underscores the importance of addressing the challenges in conducting clinical trials in Saudi Arabia through targeted educational programs, training initiatives, and enhanced support from the SNIH. By implementing strategies that focus on funding, resources, training, and collaboration, Saudi Arabia can significantly advance its clinical trial landscape, improve research quality and efficiency, and position itself as a leader in global health research.

## 1. Introduction

Clinical trials are essential research studies that aim to evaluate new medical tests and treatments to determine their effectiveness in improving human health outcomes [1]. These trials involve a series of activities designed to test hypotheses and generate conclusions that can be applied broadly to enhance our understanding of human health and diseases. The ultimate goal of clinical research is to improve medical care, public health, and to develop safe and effective interventions for the prevention, diagnosis, and treatment of various diseases [2].

Given the diverse genetics of populations worldwide, it is crucial to conduct clinical trials specifically tailored to the Saudi population. Advanced research on medical interventions for the personalized medicine of Saudi individuals is a bold effort to enhance healthcare services and reduce the rates of morbidity and mortality in the region. Clinical trials play a pivotal role in Saudi Arabia, with the potential to significantly impact the country’s pharmaceutical industry, future economic growth, and the well-being of patients. These trials not only have the power to shape the nation’s global presence but also offer Saudi Arabia a unique opportunity to establish a strong research foundation and excel in the international market.

**Figure 1:**
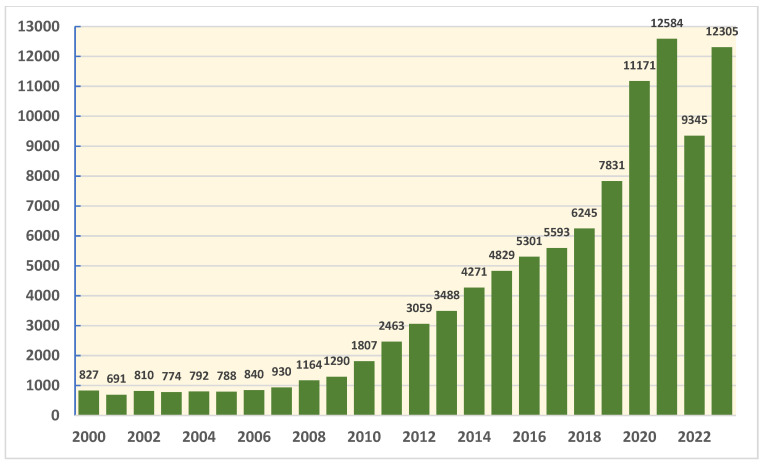
Quantitative analysis of scientific publications from Saudi Arabia in health sciences from 2000 to 2023: InCites—Clarivate, March 2024.

**Figure 2:**
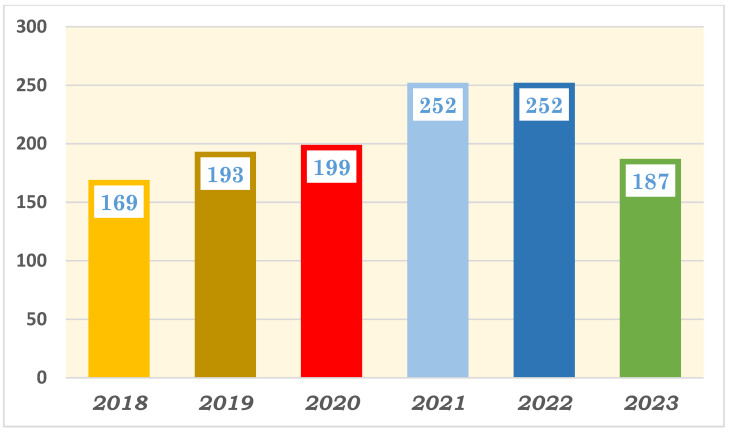
Quantitative analysis of clinical trial publications with coauthors from Saudi Arabia from 2018 to 2023: PubMed literature review in July 2024.

The health sector in Saudi Arabia has made significant improvements in research productivity in recent years, driven by factors such as increased government funding, capacity building programs through student scholarships abroad, collaborations with international institutions, improvements in research infrastructure, and a growing emphasis on evidence-based healthcare policies. Hence, Saudi Arabia has experienced a steady increase in the number of research publications in recent years. Figure 1 presents the number of scientific publications from Saudi Arabia from 2000 to 2023; the data were gathered by our team from InCites, Clarivate, in March 2024. As demonstrated in the figure, the number of research publications from Saudi Arabia has more than doubled in the last decade, reflecting the growing emphasis on research and innovation in the country’s health sector. However, it is concerning that there has been a stagnant rate of clinical trial productivity in Saudi Arabia over the last 6 years. Figure 2 presents the number of published clinical trials, coauthored by a researcher from Saudi Arabia from 2018 to 2023; the data were gathered by our team through a PubMed literature search in July 2024. Despite the surge in research publications, the lack of growth in clinical trials is a noteworthy issue that needs to be addressed to enhance the impact of research in the country’s health sector. This research project aimed to understand and address the challenges faced in conducting clinical trials in Saudi Arabia. By gathering insights from clinical trial professionals from different disciplines and research scientists, we hope to develop strategies that will improve the clinical trial process in the country.

Clinical trials hold a key position in the context of Saudi Arabia’s Vision 2030, highlighting the crucial role research and development play in shaping the country’s future. The emphasis on enhancing research education and clinical research has become a pivotal factor in driving progress. Recent initiatives undertaken by the Saudi National Institute of Health (NIH) have made notable contributions to the research landscape in Saudi Arabia, with cutting-edge trials and studies conducted in state-of-the-art facilities, setting a benchmark for excellence [3]. Despite these advancements, the frequency of clinical trials remains relatively low due to the challenges encountered in the process [4].

The clinical trial industry in Saudi Arabia faces various challenges stemming from the intricate regulatory landscape, evolving rules, and differences between regulatory bodies. Navigating these complexities becomes crucial, particularly in emerging markets where a comprehensive understanding of new requirements is essential. Slow patient recruitment and limited access pose significant obstacles, while issues such as clinical efficacy, uncontrollable toxicity, and poor pharmacokinetic properties further add to the challenges faced by the industry. Commercial demand and inadequate strategic planning also contribute to the overall complexity.

Moreover, the design of clinical trials has become increasingly intricate, mirroring the expanding complexity of clinical research. This complexity raises concerns about failure rates and potential delays in achieving primary endpoints. Contract negotiations and budget allocations often prove time-consuming due to factors like inexperienced staff, insufficient budget templates, and prolonged legal reviews.

Enhancing collaboration and data sharing among researchers, institutions, and sponsors can bolster the pace of clinical research. However, challenges related to data privacy, intellectual property rights, and competitive interests can impede effective collaboration and hinder the smooth flow of information in the research community.

Through this qualitative study, our research delves into the obstacles encountered by researchers and proposes recommendations to bolster clinical research and trials in the Kingdom of Saudi Arabia.

This study aims to investigate the challenges faced in conducting clinical trials in Saudi Arabia and propose recommendations for developing national strategies to enhance the country’s clinical trial capacities, while simultaneously working to improve research education and training in this domain and elevate the standards of clinical trials within the Kingdom’s healthcare system through providing educational programs.

## 2. Methodology

**Study Design:** The study design for this research project was qualitative study with grounded theory type, which aimed to develop a theory or explanation based on the data collected from participants. Grounded theory is particularly well-suited for exploring complex phenomena, such as the challenges and solutions in conducting clinical trials in Saudi Arabia, where the goal is to generate new insights and understandings from the perspectives of the participants themselves.

**Participants:** Participants in the study were clinical trials professionals from various disciplines (medicine, nursing, pharmacy, and applied health sciences), including investigators, clinical research coordinators, regulatory affairs professionals, and ethics committee members. Participants were recruited from hospitals, research institutions, and pharmaceutical companies in Riyadh, Jeddah, and Al Khobar, Saudi Arabia. A total of 75 participants were invited to take part in the study, with the aim that 50% would agree to attend. We needed at least 10 participants in each of the three workshops, with the total sample size being around 30 participants.

**Sampling Techniques:** Participants were recruited through purposive sampling, with invitations sent out to pre-determined list of clinical trials professionals in the three cities. Information about the study and workshop dates were disseminated with the invitation through professional networks and email invitations. Participants who expressed interest in the study registered to the event and further details of the workshop were sent to them by email or direct phone communication following the confirmation of their attendance. Prior to the workshops, participants were provided with detailed information about the study objectives, procedures, and data handling. They were also informed about their rights as research participants, including the voluntary nature of participation and the confidentiality of their responses. Electronic informed consent was obtained during the registration process of the day of the event and prior to the start of the workshops.

**Workshop Design:** The study involved three half-day workshops, each held in Riyadh, Jeddah, and Al Khobar. The workshops were facilitated by a trained moderator and note-taker for each focus group, both of whom are experienced in qualitative research methods. Each workshop consisted of small group discussions, with participants grouped into teams of 5–6 individuals. The groups were composed of participants from different disciplines to encourage diverse perspectives and insights. The workshop began with a pre-workshop survey which included basic demographic characteristics of the participants, with informed consent obtained at the end. Then, a welcome address was given, and all participants and research team members were introduced to create a comfortable and inclusive atmosphere for open discussions. An overview of the agenda, including the workshop objectives and process, was presented to guide participants through the session. A roundtable discussion was facilitated to allow each participant to introduce themselves, share their expertise, and express their perspectives on clinical trials in Saudi Arabia. The participants were grouped into 2 to 4 groups, with each group containing 4 to 5 experts and 1 facilitator for monitoring and recording. The workshop was structured into 8 different sessions; each session had one question to discuss for a period of 30 min, 15 min for brainstorming within each group, and 15 min for group presentations between groups to present their summary findings to capture key points, ideas, and themes discussed during the workshop through note-taking, audio recording, or visual documentation.

The questions selected for the workshops are as follows:

1. What are the skillsets required by practitioners to be competent in clinical trials research?
2. What are the challenges and barriers when conducting clinical trials research in Saudi Arabia?
3. What are the solutions and recommendations for addressing the challenges mentioned about clinical trial conduct in Saudi Arabia?
4. What are the current gaps in research education in Saudi Arabia?
5. What are the strategies that should be implemented by Saudi NIH to enable clinical trials?
6. What are the Saudi NIH roles in educational programs for clinical trials?
7. What resources do you believe are essential to conducting clinical trials and which one do you expect the SNIH will provide?
8. What initiatives should the SNIH implement to promote clinical trials and research education?

**Data Collection:** The data collected through a pre-workshop survey were considered quantitative data, and the data from focus group discussions during the workshops were considered as qualitative data. The discussions were guided by a set of open-ended questions mentioned above related to the challenges and solutions in conducting clinical trials in Saudi Arabia. Participants were engaged in a brainstorming session for 15 min to generate ideas and solutions within their small groups, which were recorded through handwritten notes by the facilitators. Following this, each group presented their key findings and recommendations to the larger group for further discussion; this was recorded and later a transcript for each question was developed, validated by other research team members, and prepared for thematic analysis.

**Data Analysis:** The data collected during the workshops consisted of both quantitative and qualitative components. Quantitative data were gathered through a pre-workshop survey, which included structured questions designed to assess participants’ experiences and perspectives on clinical trials. The survey was developed based on pre-established, validated instruments commonly used in quantitative research settings to ensure reliability and validity. In addition to the quantitative data, qualitative data were collected through handwritten notes and audio transcripts from the discussions during the workshops. These discussions captured key insights, recommendations, and challenges raised by participants, which were subsequently analyzed using thematic analysis to identify recurring themes and patterns. Thematic analysis was performed to identify patterns, trends, and commonalities in the responses from the focus group discussions. Initially, two researchers independently coded a subset of the transcripts and developed a coding framework. They applied the framework to the remaining transcripts, with regular discussions to refine the codes and reach consensus on the emerging themes. The findings were validated by the participants by sharing a summary of the key themes and seeking feedback to ensure accuracy and representation of their perspectives. A comprehensive report or summary of the workshop outcomes, detailing the key insights, recommendations, and strategies proposed was developed by the senior research scientists from the team.

**Validation techniques:** To enhance the trustworthiness of the study findings, several strategies were employed. These include member checking, where participants had the opportunity to review and provide feedback on the data interpretation; peer debriefing, where the research team discussed the interpretation of the data with other colleagues to ensure rigor and reliability; and reflexivity, where researchers reflected on their own biases and assumptions throughout the data analysis process.

**Ethical Considerations:** This study (study number SPJ24/011/7) was approved by the Institutional Review Board Committee at King Abdullah International Medical Research Center (KAIMRC). The study adhered to ethical guidelines for research involving human participants. In addition to obtaining informed consent, the study ensured participant confidentiality by removing all identifying information from the transcripts and using pseudonyms in any reporting of the findings. Participants were aware of their right to withdraw from the study at any time without penalty.

## 3. Results

### 3.1. Characteristics of Study Participants

**Figure 3:**
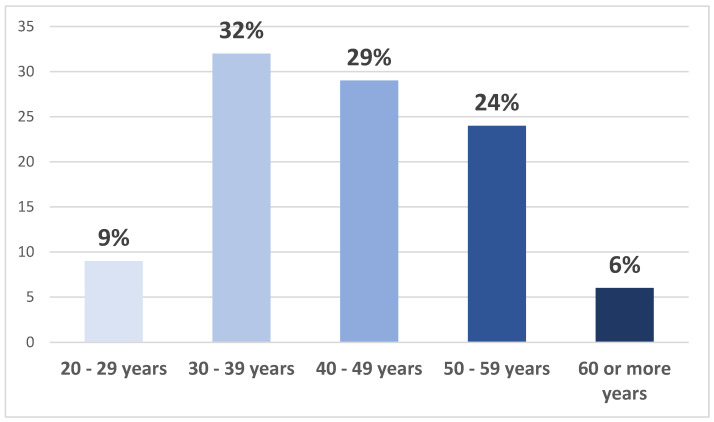
Age distribution of study participants.

A total of 35 participants attended the three workshops held in Riyadh, Jeddah, and Al Khobar, representing a diverse range of sectors. The majority of participants (twenty-three participants, 67%) were from the government sector, followed by four participants (12%) from the private sector, three participants (9%) from the semi-government sector, two participants (7%) from non-profit organizations, and two participants (5%) from other sectors. In terms of profession, physicians constituted the largest group with eleven participants (31%), followed by six research scientists (17%), five pharmacists (14%), three participants from nursing (9%), two participants from applied health sciences (6%), and eight participants (23%) from other professions. The participants were predominantly Saudi nationals, with twenty-nine participants (83%) being Saudi and six participants (17%) being non-Saudi. Additionally, 23 participants (65%) held academic positions and were engaged in clinical practice. In terms of gender, the participant breakdown was 47% male (16 participants) and 53% female (19 participants). The age distribution of the participants is presented in Figure 3 below.

### 3.2. Quantitative Analysis: Pre-Workshop Survey

The survey conducted before the workshop examined the study participants’ level of interest and experience in clinical trials, providing valuable insights into their perspectives and preparedness to participate in research activities of this nature. Among the participants, it was found that 74% expressed a willingness to participate in a clinical trial as a study subject. This high level of interest demonstrated the potential for recruitment and engagement of individuals in clinical research within the Saudi Arabian context. Furthermore, 76% of participants were willing to allow a family member to participate in a clinical trial, indicating a sense of trust and support for research participation within familial networks. In terms of experience in conducting clinical trials, 48% of the study participants had previous experience as an investigator in such research endeavors. This shows a significant portion of the participants have firsthand experience with the intricacies and challenges of conducting clinical trials. Additionally, 44% of the participants reported previous experience in initiating a clinical trial. This suggests a certain level of familiarity with the initial steps and processes involved in launching a clinical trial, which can be valuable in understanding the various challenges and barriers that may arise during this phase of research.

The results of our quantitative analysis revealed some interesting insights into the challenges faced in conducting clinical trials in Saudi Arabia. The perception rates for common challenges were quite high among the participants, indicating a significant level of difficulty in conducting clinical trials in the region. The most common challenge reported by the focus groups was the lack of resources, with 82% of participants identifying this as a major obstacle. This highlights the need for more investment in research infrastructure and funding to support clinical trials in Saudi Arabia. Additionally, 82% of participants also highlighted the lack of infrastructure as a major challenge, further emphasizing the need for improved facilities and technological support for clinical trials.

Furthermore, 80% of participants identified lack of time as a significant challenge in conducting clinical trials, indicating the need for more efficient processes and improved time management strategies. Lack of funding was also a major concern, with 80% of participants reporting this as a significant obstacle that hinders the progress of clinical trials in Saudi Arabia. The rest of common challenges are illustrated in Table 1, providing a comprehensive overview of the key obstacles faced by researchers and organizations in conducting clinical trials in the region.

In terms of the current satisfaction with the educational programs offered by national institutions, it was found that 22% of participants were not satisfied, while 27% were slightly satisfied, 32% were moderately satisfied, 18% were very satisfied, and only 2% were extremely satisfied. This indicates that a considerable portion of participants felt dissatisfied with the current educational programs available to them. Furthermore, when asked to rate their perception of the quality of these educational programs, 12% considered them to be poor, 45% labeled them as average, 35% rated them as good, and only 8% deemed them excellent. This suggests that there is room for improvement in the quality of educational programs offered by national institutions in Saudi Arabia.

**Figure 4:**
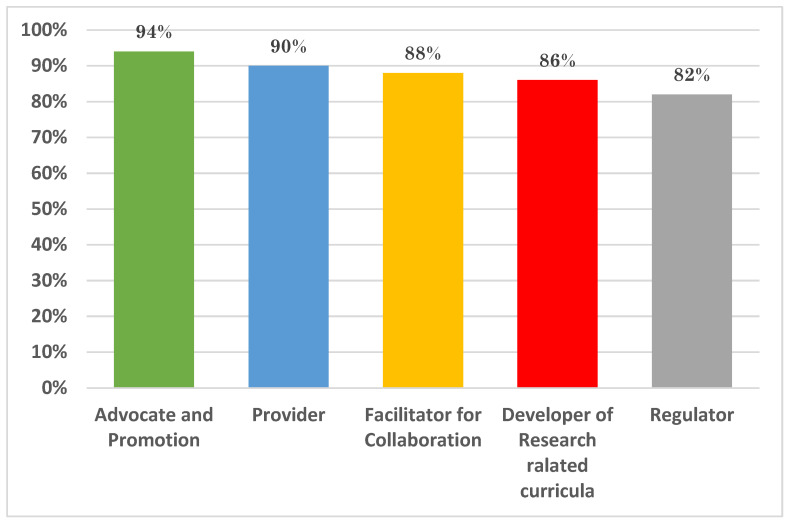
The participants’ perception of the suggested role of the Education and Research Skills Directory at the Saudi National Institute of Health.

Regarding the effectiveness of current research resources, many participants (28%) rated them as poor, while 40% considered them to be average, 23% rated them as good, and only 8% deemed them excellent. This indicates that there are significant challenges in accessing effective research resources in Saudi Arabia. Figure 4 displays the results by participants when they were asked about their perception of the role of the Education and Research Skills Directory at the Saudi National Institute of Health.

### 3.3. Qualitative Analysis: The Focus Groups Workshops

The qualitative analysis of the focus group sessions provided valuable insights into the challenges and solutions in conducting clinical trials in Saudi Arabia. The analysis revealed common themes that emerged across the eight different sessions, with one open-ended question per session.

#### 3.3.1. Question 1: What Are the Skillsets Required by Practitioners to Be Competent in Clinical Trials Research?

The respondents highlighted several core skills necessary for practitioners to be competent in clinical trials research. Fundamental research skills such as understanding statistics, conducting literature reviews, medical writing, and basic project management were emphasized. One respondent noted, “It is essential for practitioners to have a strong foundation in statistics, medical writing, and literature review to conduct meaningful research”, a statement echoed 45 times. Additionally, specific knowledge in clinical trials, such as Good Clinical Practice (GCP) and clinical trial protocols, was frequently mentioned. “Knowledge of Good Clinical Practice (GCP) and clinical trial protocols is crucial for the successful conduct of trials”, stated another respondent, with this requirement being mentioned 52 times. Effective leadership and project management skills were also deemed vital for overseeing clinical trial operations and managing research teams efficiently. “Effective leadership and project management skills are necessary to oversee clinical trial operations and manage research teams efficiently” was a common theme mentioned 34 times. Table A1 summarizes the themes and the key findings for the skillsets required to be competent researcher in clinical trials.

#### 3.3.2. Question 2: What Are the Challenges and Barriers When Conducting Clinical Trials Research in Saudi Arabia?

A significant barrier identified by respondents is the complex regulatory and bureaucratic landscape. The approval process for clinical trials was often described as too lengthy and involving multiple layers, which delays the initiation of trials. “The approval process is too lengthy and involves multiple layers, which delays the initiation of clinical trials”, explained a respondent, a concern mentioned 29 times. Additionally, the lack of unified consent forms complicates the regulatory process, creating further hurdles. “Lack of unified consent forms complicates the regulatory process and creates additional hurdles”, noted another respondent, with this issue being raised 17 times. Securing funding for clinical trials emerged as another major challenge, with respondents highlighting the need for more government and institutional support. “Securing funding for clinical trials is a major challenge, and there is a need for more government and institutional support”, emphasized a respondent, a challenge highlighted 67 times. Furthermore, a notable lack of necessary infrastructure to conduct high-quality clinical trials, such as certified labs and data management systems for efficient data collection was mentioned. “We lack the necessary infrastructure to conduct high-quality clinical trials, such as certified labs and data management systems”, stated a participant, an issue raised 40 times. Table A2 summarizes the themes and the key findings for the challenges and obstacles in clinical trial conduction.

#### 3.3.3. Question 3: What Are the Solutions and Recommendations for Addressing the Challenges Mentioned About Clinical Trial Conduct in Saudi Arabia?

Several recommendations emerged to address the identified challenges and enhance the conduct of clinical trials in Saudi Arabia. Developing national educational initiatives to provide comprehensive training in research methodology, clinical trial conduct, and regulatory requirements was seen as crucial. “Developing national educational initiatives to provide comprehensive training is crucial”, noted one respondent, a sentiment mentioned 36 times. Additionally, consolidating clinical research committees and streamlining approval processes to reduce bureaucratic delays was essential. “Streamlining regulatory processes will help reduce delays in clinical trials”, explained another participant, a view shared 29 times. Securing government and institutional funding and investing in infrastructure to support clinical research were necessary steps. “Increased funding and better infrastructure will significantly improve clinical trials”, emphasized a respondent, a recommendation mentioned 34 times. Launching public education campaigns and developing a centralized website in Arabic to provide information on ongoing clinical trials and their outcomes was suggested to enhance public awareness. “Public education campaigns and a centralized information hub are vital for increasing awareness”, stated another participant, a recommendation mentioned 31 times. Establishing dedicated clinical trial units within hospitals and research institutions and providing advanced training in project management, budgeting, and contracting were also recommended. “Dedicated clinical trial units and advanced training will enhance operational efficiency”, noted a respondent, a suggestion mentioned 28 times. Table A3 summarizes the suggested solution and recommendations to enable clinical trial conduct in Saudi Arabia.

#### 3.3.4. Question 4: What Are the Current Gaps in Research Education in Saudi Arabia?

There are several gaps in research education in Saudi Arabia that need to be addressed. Respondents frequently mentioned a lack of comprehensive training programs that cover essential aspects of clinical trials and research methodologies. “There is a need for comprehensive training programs that cover essential aspects of clinical trials and research methodologies”, noted one participant, a gap mentioned 40 times. Additionally, the lack of practical, hands-on training opportunities was highlighted. “We need more practical, hands-on training opportunities to develop competent researchers”, emphasized another respondent, a need mentioned 35 times. The absence of mentorship programs to guide new researchers was also pointed out. “There is an absence of mentorship programs to guide new researchers”, stated a participant, a gap mentioned 30 times. Furthermore, there is insufficient emphasis on the ethical aspects of clinical trials in current training programs. “Current training programs do not sufficiently emphasize the ethical aspects of clinical trials”, noted a respondent, a concern raised 25 times. Table A4 summarizes the themes and key findings of the current gaps in research education in Saudi Arabia.

#### 3.3.5. Question 5: What Are the Strategies That Should Be Implemented by Saudi NIH to Enable Clinical Trials?

To enable clinical trials, the Saudi NIH should implement several strategies. Developing and standardizing training programs that provide comprehensive education on clinical trial methodologies, regulatory requirements, and ethical considerations is crucial. “The Saudi NIH should develop and standardize training programs to provide comprehensive education on clinical trial methodologies”, suggested one respondent, a recommendation mentioned 38 times. Additionally, creating a centralized regulatory framework to streamline the approval process and reduce bureaucratic delays was frequently mentioned. “Creating a centralized regulatory framework will streamline the approval process and reduce delays”, noted another participant, a strategy mentioned 33 times. Establishing funding regulations to support clinical trials and research initiatives was also deemed necessary. “Establishing funding mechanisms to support clinical trials is essential”, emphasized a respondent, a recommendation mentioned 37 times. Promoting public engagement and awareness through education campaigns and partnerships with the media was suggested to enhance community involvement in clinical trials. “Promoting public engagement through education campaigns will enhance community involvement”, stated a participant, a strategy mentioned 32 times. Lastly, fostering collaborations with international research organizations to share knowledge and resources was highlighted as beneficial. “Fostering collaborations with international research organizations will benefit our clinical trials”, noted a respondent, a suggestion mentioned 30 times. Table A5 summarizes the suggested strategies to enable the clinical trial ecosystem in Saudi Arabia.

#### 3.3.6. Question 6: What Are the Saudi NIH Roles in Educational Programs for Clinical Trials?

The Saudi NIH plays a crucial role in developing and implementing educational programs for clinical trials. Respondents frequently mentioned the need for the NIH to provide comprehensive training in clinical trial methodologies, regulatory requirements, and ethical considerations. “The Saudi NIH should provide comprehensive training in clinical trial methodologies and ethical considerations”, suggested one participant, a role mentioned 36 times. Additionally, the NIH should establish mentorship programs to guide new researchers and provide practical, hands-on training opportunities. “Establishing mentorship programs and providing hands-on training opportunities are essential roles for the Saudi NIH”, emphasized another respondent, a role mentioned 32 times. The NIH should also promote interdisciplinary collaboration and create platforms for knowledge exchange among researchers. “Promoting interdisciplinary collaboration and creating platforms for knowledge exchange are important roles for the NIH”, noted a participant, a role mentioned 30 times. Furthermore, the NIH should ensure that training programs emphasize the ethical aspects of clinical trials. “Ensuring that training programs emphasize ethical considerations is a critical role for the NIH”, stated a respondent, a concern raised 28 times. Table A6 summarizes the themes and key findings for the Saudi NIH roles in educational programs for clinical trials.

#### 3.3.7. Question 7: What Resources Do You Believe Are Essential to Conducting Clinical Trials and Which One Do You Expect the SNIH Will Provide?

Respondents identified several essential resources for conducting clinical trials, which they expect the Saudi NIH to provide. Funding was frequently mentioned as a critical resource necessary for supporting clinical trial activities. “Funding is a critical resource necessary for supporting clinical trial activities”, emphasized one participant, a need mentioned 45 times. Additionally, the lack of availability of certified labs and advanced data management systems was highlighted. “We need certified labs and advanced data management systems to conduct high-quality clinical trials”, noted another respondent, a resource mentioned 40 times. The presence of dedicated and trained staff to manage clinical trial operations was also deemed crucial. “Having dedicated and trained staff is essential for the smooth operation of clinical trials”, stated a participant, a need mentioned 38 times. Furthermore, respondents expected the Saudi NIH to provide regulatory support and streamline approval processes to reduce bureaucratic delays. “We expect the Saudi NIH to provide regulatory support and streamline approval processes”, noted a respondent, a resource expected 37 times. Access to comprehensive training programs and mentorship opportunities was also identified as an essential resource. “Access to comprehensive training programs and mentorship opportunities is crucial”, emphasized another participant, a need mentioned 35 times. Table A7 summarizes the themes and key findings for the resources essential to conducting clinical trials.

#### 3.3.8. Question 8: What Initiatives Should the SNIH Implement to Promote Clinical Trials and Research Education?

To promote clinical trials and research education, the Saudi NIH should implement several key initiatives. Developing national educational initiatives to provide comprehensive training in research methodology, clinical trial conduct, and regulatory requirements was seen as crucial. “Developing national educational initiatives to provide comprehensive training is crucial”, noted one respondent, a sentiment mentioned 36 times. Additionally, launching public education campaigns and developing a centralized website in Arabic to provide information on ongoing clinical trials and their outcomes was suggested to enhance public awareness. “Public education campaigns and a centralized information hub are vital for increasing awareness”, stated another participant, a recommendation mentioned 31 times. Establishing dedicated clinical trial units within hospitals and research institutions and providing advanced training in project management, budgeting, and contracting were also recommended. “Dedicated clinical trial units and advanced training will enhance operational efficiency”, noted a respondent, a suggestion mentioned 28 times. Promoting interdisciplinary collaboration and creating platforms for knowledge exchange among researchers was also highlighted. “Promoting interdisciplinary collaboration and creating platforms for knowledge exchange are important initiatives”, noted a respondent, a suggestion mentioned 30 times. Lastly, fostering collaborations with international research organizations to share knowledge and resources was deemed beneficial. “Fostering collaborations with international research organizations will benefit our clinical trials”, noted a respondent, a suggestion mentioned 30 times. Table A8 summarizes the themes and key findings for the initiatives that should be implemented to promote clinical trials and research education.

The qualitative analysis provided key insights into the challenges and skills needed for conducting clinical trials in Saudi Arabia. Participants emphasized the importance of foundational research skills, including proficiency in statistics, conducting literature reviews, medical writing, and project management. Additionally, in-depth knowledge of clinical trial protocols, particularly Good Clinical Practice (GCP), was seen as critical. Leadership and project management were also recognized as vital for efficiently overseeing clinical trials and managing research teams. Moreover, challenges such as securing adequate funding, navigating complex regulatory environments, and the lack of necessary infrastructure, such as certified laboratories and data management systems, were highlighted as significant barriers to the effective execution of clinical trials.

To address these obstacles, several recommendations were made. Simplifying the regulatory approval process, particularly by establishing a centralized IRB system, was identified as crucial for reducing delays. Enhancing governmental and institutional funding, alongside investing in infrastructure, was also emphasized as essential to overcoming resource limitations. Additionally, respondents recommended the development of comprehensive training programs, mentorship opportunities, and public education initiatives to strengthen research capabilities. Finally, fostering collaboration between institutions and creating centralized platforms for knowledge sharing were suggested as key strategies to enhance the clinical trial ecosystem in Saudi Arabia. Some characteristic findings include the following:

- Mastery of research methodologies, GCP protocols, and project management is essential for clinical trial professionals.
- Complex and protracted regulatory approval processes, coupled with a lack of unified consent forms, cause delays in trial initiation.
- The absence of adequate infrastructure, including certified labs and advanced data management systems, impedes trial quality.
- A lack of comprehensive research education and mentorship hinders the development of essential skills.
- Strengthening collaboration, both domestically and internationally, is vital for improving clinical trial efficiency and resource sharing.

In Appendix A. (Analysis), we provide more detailed insights and evidence for our research findings.

## 4. Discussion

Clinical trials are at the backbone of medical research, playing a vital role in advancing our knowledge of diseases, treatments, and patient care. They serve as the gateway to introducing new and enhanced treatments to patients. Each trial is carefully designed and executed to delve into the effectiveness, safety, and potential side effects of novel medications, medical devices, therapies, and procedures. While the design of each trial may vary, they all adhere to fundamental principles to ensure accuracy and reliability of results. Engaging in clinical trials provides patients and volunteers with an opportunity to contribute to groundbreaking discoveries and advancements in the field of medical science.

Through stringent regulations, rigorous testing, and meticulous analysis, clinical trials generate valuable data and evidence that help guide healthcare professionals in making informed decisions regarding the most effective course of treatment for their patients. Although participation in a clinical trial may present its own challenges and uncertainties, the potential benefits far outweigh the risks.

The diverse representation of sectors and professions among the workshop participants provided a broad range of perspectives on the challenges and solutions in conducting clinical trials in Saudi Arabia. The predominance of government sector attendees highlights the importance of engaging with key stakeholders in the healthcare system to address these issues. Additionally, the significant number of participants engaged in clinical practice suggests a strong interest in improving the clinical trial process among those directly involved in patient care. The near-equal gender representation also ensured a balanced representation of perspectives in addressing the challenges faced in conducting clinical trials in Saudi Arabia.

The interest and experience in clinical trials among the study participants provided a solid foundation for our qualitative analysis and discussions on the challenges and solutions in conducting clinical trials in Saudi Arabia. Their perspectives and insights undoubtedly enriched our understanding of the context-specific factors that impact the successful implementation of clinical research within the region.

The results from the pre-workshop survey point to the pervasive nature of the challenges in the clinical trial landscape in Saudi Arabia. The findings from our quantitative analysis underscore the importance of addressing the common challenges (Table 1) in order to facilitate the smooth and successful conduct of clinical trials in Saudi Arabia. By investing in resources, infrastructure, time management, and funding, stakeholders can work together to overcome these obstacles and improve the clinical trial environment in the region.

The results of the pre-workshop survey revealed valuable insights into the perception of participants on the current educational programs and the role of the Education and Research Skills Directory at the Saudi National Institute of Health. These findings highlight the need for concerted efforts to enhance the quality and effectiveness of educational programs and research resources in Saudi Arabia, as well as to ensure that the Education and Research Skills Directory plays a more proactive role in supporting the research skill development of participants.

In the qualitative part of the study, identifying the challenges and barriers faced by health professions in conducting clinical trials was the main purpose of our study, which was the main focus of the second question during the group’s discussion.

One of the most prominent barriers identified is the lack of infrastructure, which has been a recurring concern in previous research; previous studies have stated the unavailability of infrastructure as one of the major concerns of researchers, where the lack of policies and improvement of these procedures created a barrier [5]. Another study conducted in the United States also mentioned the lack of local supportive infrastructure as a challenge in participating in clinical research faced by clinical cardiovascular investigators [6]. Participants emphasized that the absence of adequate facilities and supportive infrastructure poses a significant hurdle to conducting effective clinical trials. For instance, one participant noted, “If I need a lab, I have to talk with my relative in another hospital because they have a lab”. This lack of available resources forces researchers to seek external support, leading to delays and inefficiencies [7].

Furthermore, the lengthy Institutional Review Board (IRB) processes and the absence of a centralized IRB system were highlighted as major obstacles. The need for centralizing IRB procedures to reduce approval times is critical, as echoed by other studies [8,9].

Another challenge is the lack of collaboration between institutions, both nationally and internationally. Participants stressed the importance of encouraging networking among practitioners and specialists at different institutions and health sectors. They recommended increased collaboration between the Ministry of Health and different stakeholders, including educational institutions, to develop robust clinical trial programs and offer intensive educational and training courses.

A significant barrier to conducting clinical trials in Saudi Arabia is the lack of financial support, a challenge that has been documented in multiple studies [8,10,11]. The absence of a clear financial aid structure and compensation system diminishes researchers’ motivation to engage in clinical trials. One participant articulated this concern: “Most people are motivated by money; having a national policy for compensation, including covering publication fees, would significantly boost research activity”.

Access to data and technological resources is another major obstacle, aligning with findings from prior research [12]. Researchers often encounter difficulties in accessing data due to complex ownership issues and stringent privacy regulations, a problem also noted in other studies [13]. Simplifying data access and improving technological support are essential steps toward overcoming these challenges.

Regulatory and ethical challenges were frequently mentioned by participants, highlighting the complexities of navigating the regulatory environment in Saudi Arabia. These concerns are consistent with findings from other research [10,14]. The lengthy and multifaceted regulatory approval process significantly delays the initiation and completion of clinical trials. Participants emphasized the need for streamlined regulatory procedures and more educated ethics committees to mitigate these delays. Djurisic, S et al. reported similar issues faced by our scientists [11]. The issues include (a) approval by multiple ethics committees with different sets of requirements leading to multiple trial contracts, and (b) a lack of well-educated ethics committees that may delay approval and regulatory assessments.

The quality and credibility of research data are also critical challenges in conducting clinical trials in Saudi Arabia. Participants expressed concerns about the lack of quality standards, which hinders scientific progress and leads to challenges in publishing valid results. As one participant noted, “I don’t know what happens behind the scenes; this affects the quality of the data”. As mentioned in other studies, the lack of quality standards was an obstacle to scientific progress, creating challenges in publishing, and allowing the reporting of false results [14]. Establishing stringent quality controls and ensuring transparency in research processes are vital to improving the credibility and outcomes of clinical trials [14].

Patient and community engagement is another significant challenge, as successful clinical trials depend heavily on participant involvement. Participants in this study highlighted issues such as resistance to new interventions on humans, lack of communication and motivation, and insufficient incentives for doctors, which have all been acknowledged as known barriers in other studies [15]. Building trust between doctors and patients is essential for increasing participation in clinical trials. As one participant mentioned, “When you gain the trust of your patient, it is much easier to convince them to participate in a trial, this trust is invaluable”. Therefore, we want to achieve the participants’ and community’s trust in clinical trials, to improve recruitment and participation rate.

Effective project management and operational oversight are crucial for the success of clinical trials. Participants noted the importance of strong leadership and efficient management practices, which are often lacking. As observed in other research, the absence of robust project management skills and clear planning and control systems can lead to the failure of clinical trials [16]. Similar findings were documented in other research which reported the lack of project management practices, failure of planning and control systems, and the absence of a leader who organizes management poses a major barrier to successfully conducting a clinical trial [16]. Addressing these management challenges is essential for improving the overall execution of clinical trials in Saudi Arabia.

**Figure 5:**
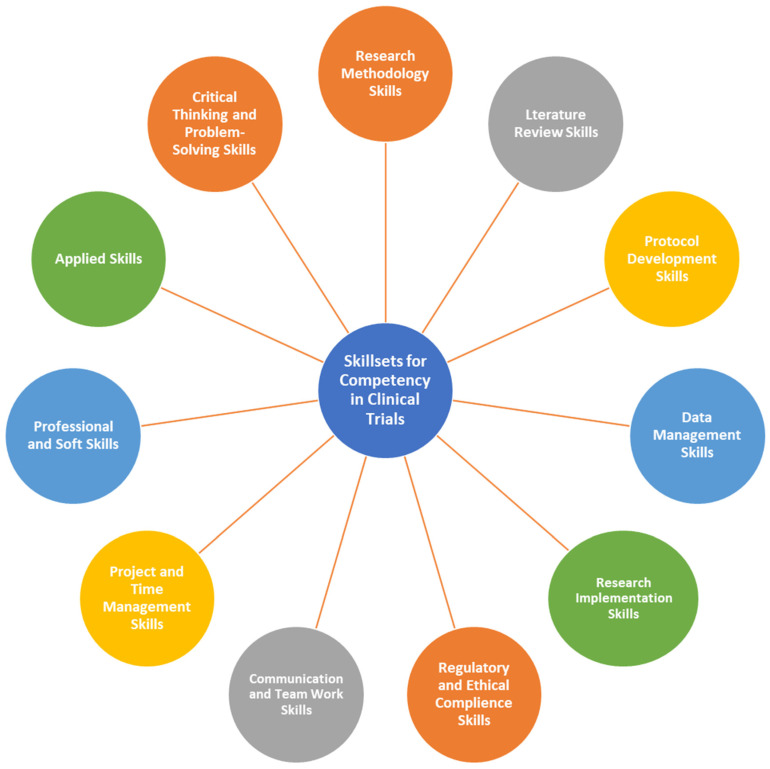
Suggested domains of skillsets required to become competent in clinical trial conduct.

In the beginning of the workshop, we explored a stimulating question: What specific skillsets are necessary to excel in conducting clinical trials? During the discussion, the participants explored what current and future skillsets are necessary for success in the clinical trial field. The majority of them are experienced professionals in this area and provided a diverse range of skills, as outlined in Table A1. The team listened to feedback and combined it with essential research skills, such as conducting literature reviews and other skills required in the near future [17,18]. They then organized these skills into 11 distinct competency domains, as shown in Figure 5. Ultimately, they determined that to excel as a clinical trial professional, one must have a wide-ranging set of abilities across multiple domains. Table A9 shows the competency required in each skillset domain in clinical trials.

Understanding research methodologies and study designs is essential to laying a strong foundation for conducting clinical trials effectively. This knowledge serves as the backbone for designing research studies, setting objectives, and ensuring analytical and critical thinking throughout the process.

Moving on to the next domains, expertise in conducting literature reviews, critically appraising research, and proficiency in data analysis, interpretation, synthesis, and presentation are crucial for gathering evidence, analyzing data, and effectively communicating research findings. These domains also encompass the ability to understand statistical results and align research activities with the overall vision and objectives.

When it comes to ethics and regulations, having a strong knowledge of research ethics, regulations, and best practices is crucial for running clinical trials ethically and following rules on both a national and global scale. Being certified in GCP (Good Clinical Practice) is also essential in this field. It is important for researchers to show ethical behavior, honesty, and accountability in all their research endeavors. The participants in the focus groups emphasized the importance of researchers truly understanding good clinical practice, rather than just obtaining certification for the sake of compliance. It seems that many currently certified researchers may not fully grasp all the concepts involved in GCP, which is a concerning issue.

In terms of communication and teamwork, excellent written and verbal communication skills, the ability to work effectively in a team, and strong interpersonal and leadership skills are essential for collaboration, coordination, and facilitating multidisciplinary teamwork. Moreover, proficient time management, task organization, and resource management skills support efficient and effective research project management.

Furthermore, possessing soft skills like leadership, collaboration, a goal-driven approach, creativity, and curiosity, along with an audit mindset, grant writing abilities, medical writing skills, research management awareness, and practical experience in conducting research, contribute to a well-rounded skillset for a clinical trial professional. Being agile, flexible, open to feedback and mentorship, and committed to ongoing professional development are also key attributes in this domain.

Applied skills and critical thinking domains like utilizing AI as a research tool, problem-solving, critical, logical, and creative thinking skills are essential for addressing challenges, finding innovative solutions, and embracing new ideas in the dynamic field of clinical research. Overall, passion, dedication, and commitment to research, combined with a comprehensive skillset spanning multiple domains, are vital for success as a clinical trial professional [17,18].

**Figure 6:**
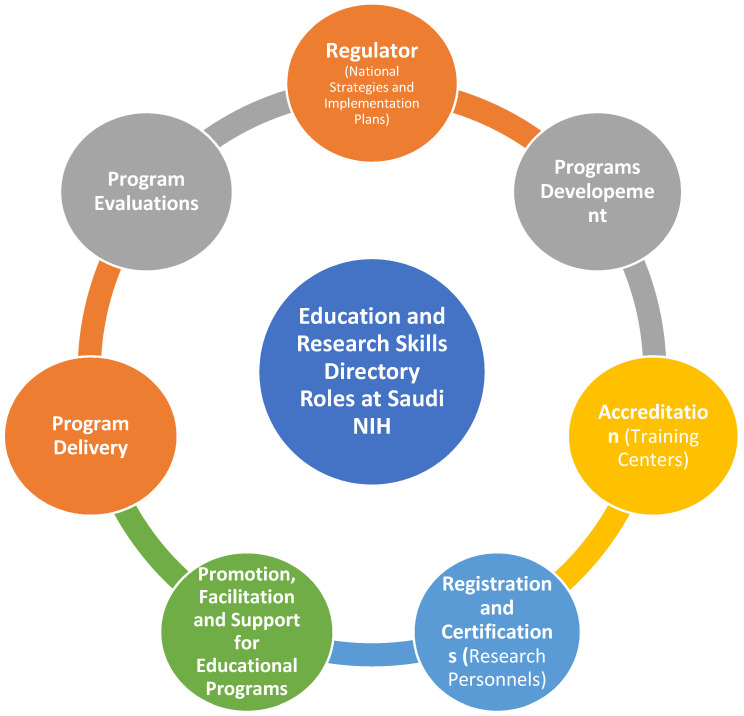
Suggested roles of the Education and Research Skills Directory and the Saudi National Institute of Health.

In the pre-workshop survey, we suggested five core roles for the Education and Research Skills Directory as shown in Figure 6. Through the focus groups, the participants suggested that there are in fact seven crucial roles that the directory could fulfill in order to support research and promote research education, as shown in Figure 6.

These roles include becoming a regulatory body in research education, working together with other stakeholders to establish national strategies for research education and oversee their implementation. Program and curriculum development would focus on creating a standardized program that can be implemented by various institutions. The directory would also be responsible for developing an accreditation process for institutions to ensure they are equipped to provide quality training programs, as well as accrediting specific programs to maintain high standards.

Another key role would be to create a platform for research registration and certification, allowing for the assessment of current staff skills, identification of skill gaps, and development of pathways for career advancement through continuous professional development programs. The directory would also be tasked with promoting, facilitating, and supporting the field of research, attracting young talent and providing necessary resources for early career scientists. Additionally, the directory would deliver specialized certification programs in collaboration with national and international centers of excellence to enhance existing skills and cultivate new ones for the future. Evaluating the effectiveness of other research education programs and conducting a national assessment of graduates from different institutions is another suggested role to ensure program objectives are being met and quality is maintained.

**Figure 7:**
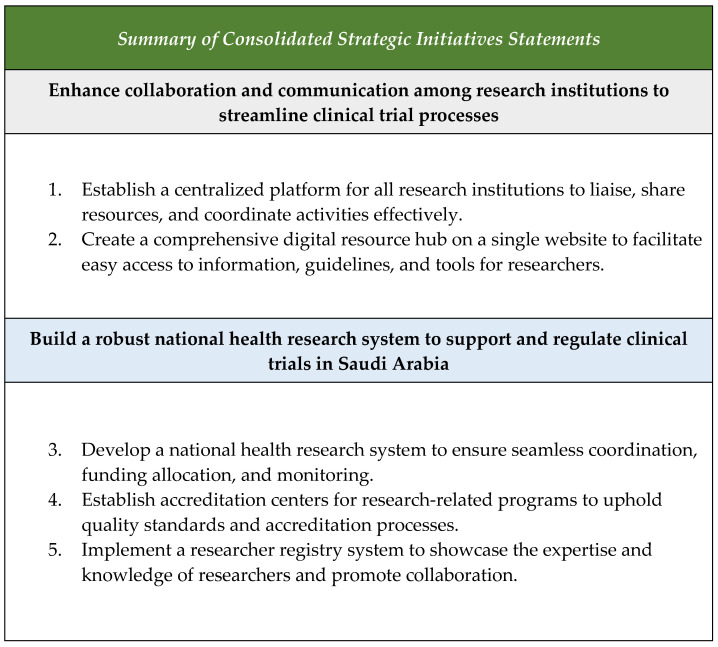

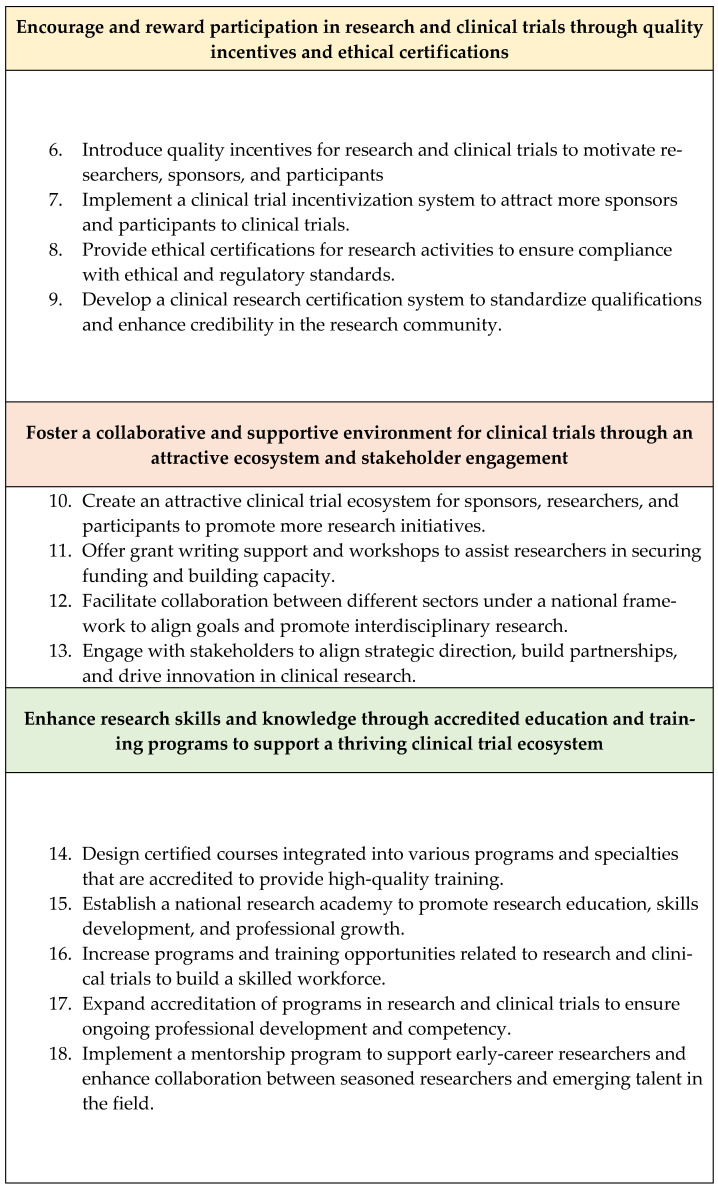
Summary of the most important strategic initiatives suggested by the participants.

**Figure 8:**
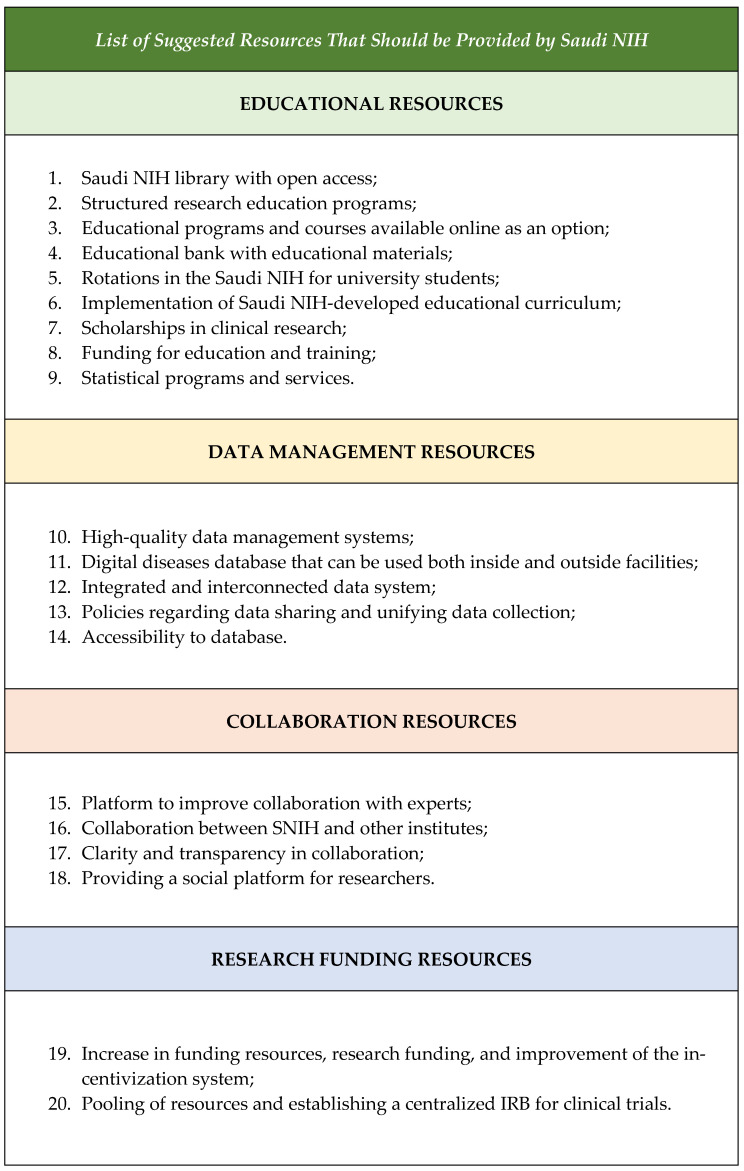
List of suggested resources that should be provided by the Saudi NIH.

The participants during the focus groups in questions 7 and 8 suggested the most important strategic initiatives that the Saudi NIH should deliver as soon as possible to develop a healthy ecosystem that enables clinical trials, as shown in Figure 7. Within those initiatives, it was obvious that research education and training should be prioritized to ensure the continued growth and success of the research community. By implementing structured training programs, mandatory research courses, and access to resources, we can empower students to excel in their research endeavors. Investing in research infrastructure, mentorship programs, and interdisciplinary collaboration will further enhance the quality and impact of research activities. Through these initiatives, we can support the development of skilled and informed researchers who will drive innovation and progress in their respective fields. Additionally, the participants emphasized the importance of having various resources easily accessible through the Saudi NIH to support the professional development of research personnel and equip them with the skills needed for future success, as shown in Figure 8.

## 5. Study Limitations

Qualitative research is a valuable tool for gaining in-depth insights into the barriers and challenges faced in clinical trials, as well as potential solutions. However, it is important to acknowledge the limitations of this approach, particularly when conducting focus groups through workshops in multiple sites. We felt that theoretical saturation was achieved as there were no more emergent themes identified by the third workshop, but this approach usually includes a smaller sample size; therefore, it limits the generalizability of the results to the general population. Probably, the most obvious weakness of this approach is the subjectivity inherent in the data collection, analysis, and interpretation of the data as it might have been influenced by the researcher’s biases and perspectives, which may have impacted the credibility and reliability of the findings.

Another significant weakness was the logistic challenges in conducting the focus groups in multiple cities. Training the research team members on how to coordinate the activities, recording the responses, making sure there was an equal participation from all members in the focus groups, and ensuring consistent facilitation across locations and across groups, may have introduced reporting bias. Participants who are professionals in the field may have varying levels of expertise and experience, which could have influenced the depth and breadth of the discussions.

## 6. Conclusions

This study suggested important recommendations and strategies for developing a supportive ecosystem for clinical trials in Saudi Arabia, highlighting the importance of investing in funding, resources, training, and collaboration across various domains. Implementing the identified recommendations can significantly advance the quality, efficiency, and impact of research initiatives in the country. By prioritizing strategies such as developing research incentives, streamlining regulatory processes, increasing funding support, and promoting standardized practices, Saudi Arabia can create a conducive environment for conducting high-quality clinical trials, fostering collaboration, and driving innovation in healthcare. These initiatives not only attract top talent and sponsors but also ensure that researchers have the tools and support needed to conduct high-quality studies. It is imperative that we continue to prioritize education, streamline processes, and enhance infrastructure to establish Saudi Arabia as a leader in conducting impactful clinical trials.

Based on the qualitative analysis of the focus group discussions, several recommendations emerged to address the challenges in conducting clinical trials in Saudi Arabia. These recommendations are categorized into four main areas: Funding and Resources, Training and Capacity Building, Performance and Collaboration, and Data and Infrastructure. Each area emphasizes the need for strategic improvements to enhance the clinical trial ecosystem and create a more conducive environment for research.

First, in the domain of **Funding and Resources**, it is critical to allocate dedicated research budgets at the hospital level to provide researchers with the necessary tools, equipment, and support. Expanding funding through the Saudi National Institute of Health (SNIH) and creating incentivization systems are key strategies to foster participation and investment in clinical research. Additionally, implementing a national reward system for research contributions, such as publications and grants, would motivate excellence and innovation. Ensuring the availability of necessary resources, including certified labs and advanced data management systems, would also be pivotal in enhancing the quality of clinical trials conducted in the Kingdom.

In terms of **Training and Capacity Building**, it is essential to develop standardized and unified research curricula aligned with national priorities. Introducing research education earlier in academic programs and offering more intensive training in key areas such as Good Clinical Practice (GCP) will ensure that future researchers are well-prepared. Establishing mentorship programs and expanding fellowship opportunities are necessary to build a skilled workforce and provide career pathways for researchers. Public awareness campaigns are also vital in addressing cultural barriers and enhancing understanding of the importance of clinical trial participation.

The **Performance and Collaboration** domain focuses on fostering greater collaboration between institutions, both nationally and internationally. This includes creating centralized regulatory bodies such as a central IRB to streamline the approval process, reducing delays in clinical trial initiation. Developing clear guidelines and policies, as well as site-level key performance indicators (KPIs), will ensure accountability and improve research outcomes. By encouraging interdisciplinary collaboration and engagement with global research platforms, the Kingdom can elevate its research capacity and visibility on the global stage.

Finally, the **Data and Infrastructure** recommendations emphasize the need for robust electronic medical record (EMR) systems and centralized platforms to streamline clinical trial data collection, storage, and sharing. Establishing a national clinical trial data registry and fostering the use of ICD-10 coding for standardized data documentation will improve the accuracy and efficiency of clinical trials. Additionally, shared hubs and collaborative spaces for researchers will facilitate knowledge exchange and innovation, creating a thriving ecosystem for clinical research in Saudi Arabia.

These comprehensive recommendations form a strategic roadmap for developing a supportive and sustainable ecosystem for clinical trials in Saudi Arabia. By addressing key gaps in funding, infrastructure, education, and collaboration, these initiatives will significantly enhance the clinical trial landscape and position the Kingdom as a leader in medical research.

## Figures and Tables

**Table 1:** The participants’ perception on the rate they encountered the common challenges of clinical trials.

The Challenge/Obstacle	Percentage of Frequently Encountered
Lack of resources	82%
Lack of infrastructure	82%
Lack of time	80%
Lack of fund	80%
Managing competing activities	78%
Lack of formal research groups	78%
Lack of personal rewards	76%
Lack of institutional support	76%
Lack of network and collaboration	76%
Lack of training programs	76%
Lack of mentorship and supervision	72%
Lack of data	72%
Poor quality of data	72%
Lack of knowledge	62%
Lack of interest from colleagues	62%
Lack of interest from trainees	62%

## Data Availability

Data can be available upon request from the research team.

## Appendix A. Analysis

**Table A1:** Summary of thematic analysis for the skillsets required to be a competent researcher in clinical trials.

Emergent Themes	Key Findings
Research Methodology and Study Design	Thorough understanding of research methodologies, study designs, and biostatistics
	Expertise in conducting literature reviews and critically appraising research
	Designing research studies and setting research objectives
	Analytical and critical thinking
Data Management and Analysis	Proficiency in data analysis
	Proficiency in data interpretation
	Proficiency in data synthesis and presentation
	Ability to understand statistical results
Regulatory and Ethical Compliance	Knowledge of research ethics, regulations, and best practices
	GCP (Good Clinical Practice) knowledge and certification
	Awareness and adherence to clinical trial regulations, nationally and internationally
	Demonstration of ethical conduct, honesty, and accountability
Communication and Collaboration	Excellent written and verbal communication skills
	Ability to work effectively in a team and as a facilitator
	Strong interpersonal and leadership skills
	Coordination and multidisciplinary collaboration
Project and Time Management	Proficient in time management, task organization, and resource management
	Ability to align research activities with the overall vision and objectives
	Documentation skills
	Agility and flexibility
Professional Development and Soft Skills	Ability to think critically, logically, and creatively
	Curiosity, willingness to think outside the box, and embrace new ideas
	Openness to feedback, mentorship, and ongoing professional development
	Passion, dedication, and commitment to research
	Soft skills like leadership, collaboration, and a goal-driven approach
Technical and Applied Skills	Research management and awareness of the local clinical trial ecosystem
	Grant writing
	Audit mindset
	Understanding of clinical trial business
	Medical writing skills
	Practical experience in conducting research
	Using AI as a research tool
Problem-solving and Critical Thinking	Skills in problem-solving and troubleshooting
	Ability to think critically and logically
	Creative thinking
	Analytical thinking

**Table A2:** Summary of the thematic analysis for the challenges and obstacles in clinical trial conduction.

Emergent Themes	Key Findings
**Organizational and Infrastructure Challenges**	Lack of experienced research teams (IRB, coordinators, biostatisticians, and clinical trial scientists)
	Lack of research sites and infrastructure
	Absence of centralized clinical trials management systems
	Lack of organized bodies to facilitate multi-center collaborations
	Lack of open data repositories and research-enabling infrastructure
**Funding and Incentives Challenges**	Lack of funding
	Shortage of human resources (financial analyst)
	Lack of rewards, recognition, and financial support for research
	Funding constraints and lack of dedicated research budgets
**Data and Technology Challenges**	Limited access to databases and patient screening tools
	Challenges with data accessibility, and integration of different EMR systems
	Challenges with data collection, analysis, and interpretation
**Collaboration and Networking Challenges**	Lack of clear collaboration pathways between research centers
	Limited collaboration and data sharing between institutions
	Cultural barriers, both internal and external to research institutions
**Regulatory and Ethical Challenges**	Complex regulatory environment with overlapping responsibilities and multilayered submissions
	Challenges with ethical considerations and regulatory approvals
**Training and Mentorship Challenges**	Lack of mentorship and guidance for researchers
	Limitations in research expertise, experience, and training
	Shortage of qualified research staff and biostatisticians
	Insufficient research knowledge and strategic prioritization
	Lack of leadership skills
**Research Quality and Validity Challenges**	Concerns about scientific validity and research quality
	Challenges with research methodology and study design quality
**Patient and Community Engagement Challenges**	Lack of community awareness and education of research subjects
	Challenges with patient recruitment and retention
	Lack of motivation for physicians and researchers
**Project Management and Operations Challenges**	Difficulties with contracting, budgeting, and supply chain management
	Lack of research output support (e.g., medical writing, finance)
	Lack of leadership and dedicated research coordinators
	No protected time for researchers

**Table A3:** Summary of the thematic analysis of the suggested solutions and recommendations to enable clinical trials in Saudi Arabia.

Emergent Themes	Key Findings
**Site and Institutional Readiness**	Develop research incentives and KPIs for sites to encourage clinical trial participation
	Implement robust electronic medical record (EMR) systems
	Allocate dedicated resources and budgets for research at the hospital level
	Implement site-level KPIs for researchers, whether conducting investigator-initiated trials (IITs) or sponsored clinical trials
	Increase the capability of research centers to run global trials
	Establishing a CTU or improving an existing one
**Education, Training, and Capacity Building**	Provide intensive education, training, and workshops to improve research culture and capacity
	Establishing a national educational framework and delivering high-quality educational services
	Become the platform for clinical research education, the only reference for research education programs accreditation, and the regulatory institution for clinical research education.
	Develop a national clinical trial mentorship program
	Conduct clinical trials education and awareness campaigns at the public and societal level to overcome cultural barriers
	Ensure principal investigators (PIs) have the necessary management skills to select and supervise their teams
	Provide comprehensive Good Clinical Practice (GCP) training for research personnel
	Recruit more qualified research staff and develop clear research career pathways
	Talent development by concentrating on selected research talents as well as technical and behavioral competencies
	Spread of regulatory requirements and policy influence
	Awarded competition
	Provide dashboard about researching by field and researching by region.
**Regulatory and Governance**	Address political barriers and improve research infrastructure
	Establish a consolidation committee at the hospital level and coordinate with national entities (SFDA, SNIH, customs, and IRB) to streamline processes
	Establish a central IRB to streamline the approval process
	Create a more attractive clinical trial ecosystem with fast-track approval timelines and smooth registration for investigational medicinal products (IMPs)
	Regulatory role and decentralization through branches to support delivering and education role.
	A unified patient consent form
**Collaboration and Visibility**	Encourage participation in international clinical trial platforms to enhance visibility and collaboration
	Establish shared hubs to facilitate collaboration among researchers
	Create a government-backed website to serve as a hub for clinical trials
	Innovation and flagship programs through collaboration with different stakeholders and organizations like pharmaceutical companies and clinical/educational entities
	Designing different research programs in collaboration with the Ministry of Education and research centers
	Collaborate with other stakeholders: academia and industry in research education
**Patient Engagement and Accessibility**	Develop readily available patient databases with user-friendly search capabilities
	Create patient advocacy groups and strengthen connections between sites and patients
**Funding and Incentives**	Increase funding for clinical research and provide dedicated marketing teams to promote research activities
	Implement a national reward system for clinical research, including support for publications and research grants
	Expand the funding available through the Saudi National Institute of Health (SNIH)
**Data and Disease Registries**	Establish a national clinical trials data registry and portal in both Arabic and English
	Connect national disease registries with SNIH databases
	Encourage researchers to learn and utilize ICD-10 coding
**Standardization**	Standardization of career paths for researchers
	Standard setting
	Encouraging health staff to standardize their research
	Standardization of policies, procedures, and guidelines for clinical trials nationally
**Strategic Planning and Innovation**	Achieve novelty and advancement in creative research
	Benchmarking with best practices globally
	Assess the needs first and then narrow the gaps
	Avoid overlap
	Assessment of all existing programs

**Table A4:** Summary of thematic analysis of the current gaps in research education in Saudi Arabia.

Emergent Themes	Key Findings
Research Curriculum and Education	Lack of a comprehensive research curriculum development
	Lack of unified research curriculum
	Lack of clinical trial education campaigns and integration within university curricula
	Shortage of research courses and training in biostatistics
	Need to introduce research education at an earlier stage (school/college)
	Lack of structured training programs
	Lack of research education as a mandatory course
	Need for more robust and standardized research curricula
Research Mentorship and Support	Lack of qualified research mentors
	Shortage of qualified professionals to teach research programs
	Lack of exposure to research and systematic education programs
	Shortage of good research mentors and mentorship culture
	Lack of training opportunities at the postdoctoral level
	Lack of nurturing critical thinking and innovation
	Lack of guidance and funding for students interested in research
Research Resources and Infrastructure	Insufficient access to research materials, equipment, and resources
	Lack of access to research facilities to undergraduate students
	Lack of well-equipped and maintained research laboratories
	Weak research governance and infrastructure
	Lack of institutional support, data maintenance, and IT resources
	Shortage of research infrastructure and experienced healthcare staff
Research Culture and Priorities	Poor quality of research education
	Emphasis on quantity and research output over quality and research impact
	Lack of emphasis on addressing local research needs
	Weak research culture and lack of time for research
	Lack of a clear national strategy for research
	Lack of transparent national priorities
Research Funding and Collaboration	Lack of funding for research
	Poor collaboration between education institutions and industry
	Challenges with accessing patient data and defining relevant research questions
	Need for research advocacy and interdisciplinary collaboration
Practical Research Training	Lack of practical, hands-on research training
	Lack of utilization of technology in research training
	Lack of training opportunities at the postdoctoral level
Research Personnel and Career Development	Shortage of well-trained or experienced research personnel
	Lack of recognition and accreditation for research professionals
	Shortage of specialized research programs for healthcare staff
	Lack of formal training, mentorship, and career path in research
	Inadequate incentives and protected time for research
Publication and Authorship	Challenges with authorship and publication considerations
	Limited exposure to research activities and conferences

**Table A5:** Summary of the thematic analysis for the suggested strategies to enable clinical trial ecosystem.

Emergent Themes	Key Findings
Governance and Policies	Develop clear guidelines and policies for clinical trials
	Simplify the approval process for clinical trials
Collaboration and Networking	Liaison between different research institutes
	Build trust and reputation with universities
	Facilitate networking and collaboration among researchers
	Enable investigators to take initiative in conducting clinical trials
	Encourage international collaboration for clinical trials
Resources and Incentives	Provide necessary resources for conducting clinical trials
	Establish a rewards system, such as national prizes, to incentivize clinical trials
	Facilitate fast-track startup timelines for clinical trials
	Provide continuous sustainable funding for clinical trials
	Implement a clinical trial incentivization system
Research Capacity Building	Provide training on how to write professional clinical trial protocols
	Establish educational pathways for researchers, such as local micro-credentials courses, diplomas, master’s, Ph.D., and fellowship programs
	Decentralize Saudi NIH operations
	Expand clinical trial-related educational and training programs
	Standardization of clinical trial curriculum development and deliveries
	Build clear career pathways for clinical trial professionals
Data and Infrastructure	Develop a patient registry for diseases, readily available for researchers in collaboration with the Ministry of Health

**Table A6:** Summary of thematic analysis for the Saudi NIH roles in educational programs for clinical trials.

Emergent Themes	Key Findings
Curriculum and Program Development	Establishing a national curriculum development committee for research education and training
	Development of research curriculum framework for implementation on a national level
	Involving research curriculum in high school and undergraduate students
	Create and fast-track the development of new clinical trial-related programs
Accreditation and Certification	Development of accreditation pathways for institutions nationally and different educational programs deliveries
	Provide a diversity of certification programs for health research
Program Facilitation, Support and Promotion	Facilitate the establishment and provision of research programs at a national level
	Increase the number of programs related to clinical research
	Provide more support for programs in different regions
	Promote research education among different stakeholders
Program Delivery	Provide certification programs in clinical trials, innovation, and knowledge translation
Stewardship and Alignment	Act as stewards for clinical trial educational programs
	Enable the development of programs and the recruitment of people to support them
	Serve as leaders in different units to support and have programs acknowledged by various entities
	Review and align clinical trial-related educational programs with national strategies
Regulatory Body for Research Education	Develop national strategy for research education
	Establish and implementation and action plans for delivery

**Table A7:** Summary of thematic analysis for the resources essential to conduct clinical trials.

Emergent Themes	Key Findings
**Educational Resources and Infrastructure**	Educational programs and course (available online as an option)
	Educational bank with educational materials
	Rotations in the Saudi NIH for university students
	Increase awareness and funding resources
	High-quality data management systems
	Knowledge self-paced programs
	Saudi NIH library (Initiative) with open access
	High-quality labs
	Budget for education and training
	Developed curriculum
**Data and Information Management**	Digital database that can be used both inside and outside facilities
	Improved data reading services
	Policies regarding data sharing and unifying data collection
	Accessibility to database and statistical services
**Collaboration and Networking**	Developing a platform to improve collaboration by allowing registration and communication with experts (e.g., biostatisticians, medical writers)
	Collaboration between the SNIH and other institutes
	Clarity and transparency in collaboration
	Providing a social platform for researchers
	Structured research education programs delivered through different learning methods
	Raising the bar for accepted research for publication
**Regulatory and Funding Support**	Pooling of resources and a centralized Institutional Review Board (IRB) for clinical trials
	Research funding, including an incentivization system
	Provision of Clinical Research Coordinators (CRCs) to needy sites
	Clearer and more consolidated research regulations
	Funding researchers and providing incentives for them
	Grants
**Manpower and Training**	IRB/GCP courses and study design
	Scholarships in clinical research
	Mentorship
	Enough and qualified staff for education and training

**Table A8:** Summary of thematic analysis for the initiatives that should be implemented to promote clinical trials and research education.

Emergent Themes	Key Findings
**Coordination and Consolidation**	Liaison and coordination between different research institutions
	Consolidation of all digital resources and information into a single website
**System and Infrastructure Development**	Establishment of a national health research system
	Establishing accreditation centers for research-related programs
	Registry system for every researcher in Saudi Arabia to distribute their expertise and knowledge
**Incentives and Certifications**	Implementing quality incentives for research and clinical trials
	Providing ethical certifications for research activities
	Implementing a clinical trial incentivization system
	Developing an identified clinical research certification system
**Ecosystem and Collaboration**	Creating an attractive clinical trial ecosystem for sponsors
	Grant writing support and workshops
	Collaboration between different sectors under a national framework
	Collaboration with concerned stakeholders
**Education and Training**	Designing certified courses that are integrated into different programs and specialties, and are accredited
	Creation of a national research academy
	Promoting research education and participation among younger audiences in schools and universities
	Increasing the number of programs and training opportunities related to research and clinical trials
	Expanding the accreditation of programs related to research and clinical trials
	Diversity in content and levels
	It should be delivered by trainers who are truly engaged in clinical research
	It should be comprehensive and inclusive of key public health priorities set by the government
	Tailored clinical research training for rural health facilities
	Online Continuing Professional Development (CPD) programs
	Have a research focus to support certain areas every once in a while
**Improvement and Analysis**	Conducting deep gap analysis to identify areas for improvement and allocating resources towards it
	Bridge challenges, distinguish loopholes, and work on them
	Build capacity and reduce gaps in the educational system
	Benchmarking
**Objectives and Standardization**	Having a clear framework that must be adjusted and tailored accordingly
	Clear guidelines and policy
	Outcomes of the strategy should be measurable and realistic
**Representation and Stakeholders**	Adequate choice of representatives
	Stakeholders’ alignment and establishing a strategic direction
	Engagement of different stakeholders
**Funding and Resources**	Sustainable funding targeting all sectors related to health research (MOH, academia, and industry)
	Appropriate funding and allowances should be provided to the trainees and trainers
	Strong funding base
**Strategic Planning**	Feedback to update strategy every few years developed based on benchmarking studies
	Key Performance Indicators (KPIs)
	Multidisciplinary involvement in developing the plan
	Outline a specific research plan to tailor all available resources to serve it
	Alignment with the country’s vision and trajectory

**Table A9:** The competency required in each skillsets domains in clinical trial.

#	Skill Domain	Skill Competency
1	Research Methodology Skills	Research methodology and study designs; Selecting appropriate research designs for clinical trials; Develop clear research objectives and hypotheses; Identifying and selecting study populations; Randomization and allocation procedures; Designing data collection and measurement methods; Outcome measures and endpoints in clinical trials; Types of biases and sources of error in study design.
2	Literature Review Skills	Literature searches using appropriate databases; Synthesizing research findings; Critically evaluate the quality and relevance of research studies; Summarizing and analyzing key findings from the literature; Cite and reference sources in accordance with academic standards; Types of literature reviews (e.g., systematic reviews); Software tools for managing literature Identify gaps in the literature; Writing literature review sections of research papers and reports; Presenting literature review findings.
3	Protocol Development Skills	Translate research questions into a clear study protocol; Writing and presenting research proposals and protocols; Designing study procedures; Drafting informed consent forms and study documents; Developing data collection instruments and case report forms; Incorporate statistical considerations into protocol design; Risks and safety concerns in the protocol; Detailed study timeline and budget; Obtaining feedback from stakeholders during protocol development; Revising and finalizing study protocols based on feedback.
4	Data Management Skills	Designing data collection forms and databases; Data cleaning and validation procedures; Data entry and processing; Data security and confidentiality protocols; Quality control checks on data; Data analysis using statistical software; Data reporting and visualization techniques; Organizing study data for analysis and reporting; Collaborating with the study team to ensure data accuracy; Handling missing or incomplete data.
5	Research Implementation Skills	Operationalize study protocols and procedures; Training study personnel on protocol procedures; Study timeline management and tracking milestones; Subject recruitment and retention strategies; Handling logistics and coordination for study activities; Troubleshooting implementation issues in real-time; Conduct site visits and monitoring visits; Budget management; Study progress tracking and reporting to stakeholders; Regulatory compliance requirements for research implementations.
6	Regulatory and Ethical Compliance Skills	Knowledge of regulatory requirements; Ethical considerations in research; Institutional Review Board (IRB) processes; Good Clinical Practice (GCP) guidelines; Informed consent process; Regulatory submissions; Adverse event reporting; Protocol adherence; Data management; Regulatory inspections and audits.
7	Communication and Teamwork Skills	Effective communication; Active listening; Interpersonal skills; Teamwork; Flexibility and adaptability; Conflict resolution; Emotional intelligence; Cultural competence; Effective interdisciplinary teams’ collaboration.
8	Project and Time Management Skills	Planning; Time management; Resource allocation; Risk management; Change management; Stakeholder engagement; Delegation; Monitoring and evaluation; Documentation; Strategic thinking.
9	Professional and Soft Skills	Empowerment; Mentorship and coaching; Motivation; Self-motivation; Accountability; Vision and inspiration; Professionalism; Confidentiality; Empathy and compassion; Collaboration. Adaptability; Leadership skills; Resilience.
10	Applied Skills	Continuous learning and professional development; Adaptability to emerging technologies, regulations, and best practices in clinical research; Technology proficiency (electronic data capture systems, remote monitoring technologies, telemedicine platforms, and digital health solutions); Digital literacy (cloud computing, wearable devices, virtual collaboration software and virtual clinical trials); Advanced data analytics tools (Artificial Intelligence); Patient-centric approaches; Digital marketing strategies; Personalized medicine approaches; Real-world evidence studies; Risk-based monitoring strategies; Virtual team management.
11	Critical Thinking and Problem-Solving Skills	Creativity; Decision-making; Problem-solving; Curiosity; Entrepreneurial mindset; Analytical skills; Logical reasoning; Critical evaluation; Attention to detail.

## Appendix B. Recommendations

The following are general recommendations to develop a supportive ecosystem for clinical trials in Saudi Arabia categorized into four main domains:

### Appendix B.1. Funding and Resources for Clinical Trial Domain

1. **Dedicated Research Budgets**
  - **Allocate dedicated resources and budgets for research** at the hospital level to ensure that researchers have the necessary tools and support to conduct high-quality studies. By investing in research infrastructure, we can attract top talent and increase the competitiveness of our site in the clinical trial landscape.
2. **Increased Funding and Marketing**
  - **Increase funding for clinical research** and establish dedicated marketing teams to promote research activities. By investing more resources into research initiatives and leveraging targeted marketing efforts, we can raise awareness about the importance of clinical research and attract more support from stakeholders and sponsors.
3. **Support from the Saudi National Institute of Health (SNIH)**
  - **Expand the funding available through the SNIH** to provide additional support for clinical research projects. By bolstering funding opportunities through the SNIH, we can create more avenues for researchers to secure financial backing for their studies and further drive advancements in health and medicine in the Kingdom.
4. **Necessary Resources for Researchers**
  - **Provide researchers with the necessary resources**, such as equipment, facilities, and personnel, to conduct their trials effectively.
5. **Funding Support for Clinical Trials**
  - **Provide funding support through grants, scholarships, or research funding programs** to enable researchers to finance their studies and ensure the sustainability of ongoing trials.
6. **Clinical Trial Incentivization System**
  - **Implement a clinical trial incentivization system**, which may include monetary rewards, access to exclusive resources, or research opportunities, to further encourage participation and investment in clinical research efforts within the Kingdom.
7. **National Reward System**
  - **Implement a national reward system for clinical research** that includes incentives for publications, research grants, and other contributions to the field. By acknowledging and rewarding researchers for their efforts and achievements, we can motivate continuous excellence in research practices and encourage innovation in the healthcare sector.

### Appendix B.2. Training and Capacity Building Domain

8. **Strengthening Research Curriculum and Promoting Clinical Trial Education Across Educational Institutions:**
  - **Develop standardized, unified, and comprehensive research curricula,** aligned with national priorities and incorporating it within university curricula and launching education campaigns. Implement research education as a mandatory course in academic curricula to ensure all students receive research training. Moreover, introduce research education at an earlier stage, starting from school or college, to foster interest and skills in research.
9. **Enhanced Training Programs and Global Partnerships**
  - **Increase the capability of research centers to run global trials** by enhancing training programs and expanding partnerships with international collaborators. By building expertise and capacity in running multi-center studies, we can position our site as a leader in conducting large-scale, impactful research projects.
10. **Intensive Education and Training**
  - **Provide intensive education, training, and workshops** to equip our researchers with the skills and knowledge needed to excel in clinical trials. Establish structured training programs with clear objectives, milestones, and mentorship support for research students. Introduce more research courses and training in biostatistics and applied skills of the future to equip students with necessary skills for research. By investing in continuous learning and development, we can enhance the capabilities of our research teams and drive innovation in our studies.
11. **Public Education and Awareness Campaigns**
  - **Conduct clinical trials education and awareness campaigns** at the public and societal level to address cultural barriers and increase understanding of the importance of research participation. By engaging with the community and raising awareness about the benefits of clinical trials, we can overcome misconceptions and encourage greater involvement in research studies.
12. **Management Skills for Principal Investigators**
  - **Ensure that principal investigators (PIs) possess the necessary management skills** to effectively select and supervise their research teams. By providing training in team leadership and project management, we can enhance the effectiveness of research teams and increase the success rate of our clinical trials.
13. **Good Clinical Practice (GCP) Training**
  - **Provide comprehensive Good Clinical Practice (GCP) training for research personnel** to uphold ethical standards and quality in research conduct. By ensuring that all research staff are well-versed in GCP guidelines, we can maintain the integrity and reliability of our study outcomes.
14. **Investigator Training Programs**
  - **Develop training programs and workshops** to equip investigators with the necessary skills and tools to take initiative in planning and conducting clinical trials, empowering them to drive progress in this area.
15. **Educational Pathways for Researchers**
  - **Establish educational pathways for researchers**, such as local master’s, Ph.D., and fellowship programs focused on clinical research, to cultivate a skilled workforce and foster expertise in conducting clinical trials within the country.
16. **Expanded Educational Programs**
  - **Expand the availability of clinical trial-related educational programs, fellowships, and Ph.D. programs** to provide researchers with diverse opportunities for advanced education and specialization in conducting high-quality clinical trials, further developing expertise and capabilities in this field.
17. **National Clinical Trial Mentorship Program**
  - **Develop a national clinical trial mentorship program** by providing training and support to qualified research mentors to guide and mentor students effectively, and to connect experienced researchers with emerging professionals. Promote a culture of mentorship in research to nurture critical thinking, innovation, and professional development among researchers. This initiative will foster mentorship relationships, facilitate knowledge sharing, and accelerate the development of future leaders in clinical research.
18. **Recruitment and Career Pathways**
  - **Recruit more qualified research staff and develop clear research career pathways** to attract and retain top talent in our organization. By investing in our research workforce and offering clear advancement opportunities, we can create a culture of excellence and drive continuous improvement in our research endeavors.
19. **Accreditation Process for Research Education Programs**
  - **Strengthen research infrastructure by developing accreditation processes** for research institutions providing training programs. Increase funding support to research institutions delivering research education and training opportunities to optimize the infrastructure to facilitate research related activities. Invest in well-equipped and maintained research laboratories, facilities, and IT resources to support research activities.
20. **Enhance access to research resources**
  - **Incorporate technology and online resources** to improve access to research training materials, data, facilities, and resources for students and researchers to enhance learning and research capabilities.
21. **Recognize research professionals**
  - **Establish registration, accreditation, and licensing processes** for research professionals to recognize and validate their skills and expertise in the field, and promote continuous professional development among research personnel.

### Appendix B.3. Performance and Collaboration Domain

22. **Research Incentives and KPIs**
  - **Develop research incentives and KPIs** to encourage site engagement in clinical trials. By aligning goals and rewarding sites for successful research outcomes, we can drive greater participation and motivation among researchers.
23. **Site-Level KPIs**
  - **Implement site-level KPIs for researchers**, whether conducting investigator-initiated trials or sponsored clinical trials, to track performance and ensure accountability. By setting clear expectations and monitoring progress, we can drive continuous improvement and excellence in research practices.
24. **Addressing Political Barriers**
  - **Address political barriers and improve research infrastructure** through strategic engagement and advocacy efforts. By collaborating with key stakeholders and policymakers, we can work towards removing barriers and enhancing support for clinical research initiatives.
25. **Consolidation Committee**
  - **Establish a consolidation committee at the hospital level** to coordinate efforts with national entities such as the Saudi Food and Drug Authority (SFDA), customs, and Institutional Review Boards (IRBs). This committee can streamline processes, reduce administrative burdens, and facilitate efficient collaborations between different entities involved in the research approval process.
26. **Central IRB**
  - **Establish a central IRB** to streamline the approval process for clinical trials. By centralizing the review process, we can ensure consistency, efficiency, and timely approvals for research studies, ultimately expediting the initiation of clinical trials.
27. **Attractive Clinical Trial Ecosystem**
  - **Create a more attractive clinical trial ecosystem** by implementing fast-track approval timelines and smooth registration processes for investigational medicinal products (IMPs). By simplifying regulatory requirements and ensuring swift approvals, we can improve the competitiveness of our site and attract more sponsors and researchers to conduct clinical trials at our institution.
28. **Clear Guidelines and Policies**
  - **Develop clear guidelines and policies for clinical trials** in Saudi Arabia, along with streamlining the trial approval process, to improve research education and training. These measures will elevate the standards of clinical trials in the country’s healthcare system and boost its capabilities in conducting trials.
29. **Fast-Track Start-up Timelines**
  - **Facilitate fast-track start-up timelines for clinical trials** by streamlining approval processes and administrative requirements. This will expedite the initiation of trials and accelerate research progress.

### Appendix B.4. Data and Infrastructure Domain

30. **Electronic Medical Record Systems**
  - **Implement robust electronic medical record systems** to streamline data collection and improve efficiency in clinical trial processes. This will enhance the quality of research conducted at our site and facilitate data sharing and collaboration with external partners.
31. **International Clinical Trial Platforms**
  - **Encourage participation in international clinical trial platforms** to enhance visibility and foster collaboration among researchers. By engaging with global networks and platforms, we can access a wider pool of expertise, resources, and opportunities for collaboration, ultimately enhancing the impact and reach of our research efforts.
32. **Shared Hubs and Collaborative Spaces**
  - **Establish shared hubs or collaborative spaces** to facilitate interactions and knowledge exchange among researchers. These hubs can serve as focal points for networking, sharing best practices, and fostering interdisciplinary collaborations, creating a vibrant ecosystem of innovation and partnership in clinical research.
33. **Centralized Hub for Clinical Trials**
  - **Create a government-backed website as a centralized hub for clinical trials** to streamline information sharing and access to research opportunities. This website can provide a comprehensive database of ongoing trials, research resources, regulatory guidelines, and funding opportunities, serving as a valuable resource for researchers, sponsors, and the public alike.
34. **Readily Available Patient Databases**
  - **Develop readily available patient databases** with user-friendly search capabilities to connect individuals interested in participating in clinical trials with relevant research opportunities. By creating easy-to-navigate databases, we can streamline the recruitment process and enhance participation rates in clinical research studies.
35. **Patient Advocacy Groups**
  - **Create patient advocacy groups** and strengthen connections between research sites and patients to foster a collaborative and supportive environment for clinical trial participants. By involving patients in the research process and amplifying their voices through advocacy groups, we can ensure that their perspectives and needs are prioritized, leading to more patient-centered research practices.
36. **National Training System for Clinical Research**
  - **Implement a national training system for clinical research** that includes promotes the excellence in research practices and encourages innovation in the healthcare sector.
37. **National Clinical Trials Data Registry**
  - **Establish a national clinical trials data registry and portal** to centralize information on ongoing clinical trials. By creating a unified platform, researchers, healthcare professionals, and the general public can access valuable information on clinical trials, facilitating transparency, collaboration, and informed decision-making.
38. **Utilization of ICD-10 Coding**
  - **Encourage researchers to learn and utilize ICD-10 coding** for accurate and standardized documentation of medical diagnoses. By incorporating ICD-10 coding practices into research protocols, we can improve data accuracy, streamline data analysis, and enhance the overall quality of clinical research studies.
